# Retraction: Triumvirate Presentation and Treatment of Psoriasis in the Setting of HIV and Treponema pallidum Infection

**DOI:** 10.7759/cureus.r56

**Published:** 2022-03-22

**Authors:** Ariel Kidron, Hiep Nguyen, Hoang Nguyen

**Affiliations:** 1 Emergency Medicine, Nova Southeastern University Dr. Kiran C. Patel College of Osteopathic Medicine, Fort Lauderdale, USA; 2 Orthopaedic Surgery, Nova Southeastern University Dr. Kiran C. Patel College of Osteopathic Medicine, Fort Lauderdale, USA; 3 Basic Sciences, Nova Southeastern University Dr. Kiran C. Patel College of Osteopathic Medicine, Clearwater, USA

This article has been retracted at the request of the authors due to incorrect authorship as detailed below:

"The work was initially divided between several contributing authors, however due to a disagreement between the authors as to which journal to publish the work in, the initial author group was broken up. Initially, after verbal communication with the team, we were under the impression that it would be ok to completely rewrite the case and publish it without some of the original authors who were in disagreement and broke away. It was a mistake to leave several of the initial authors out of the paper regardless of whether it was completely rewritten. As a result, we wish to correct that mistake and retract the article."

Cureus has reviewed this request and agreed to formally retract this article.

